# Large-Scale Qualitative and Quantitative Assessment of Dityrosine Crosslinking Omics in Response to Endogenous and Exogenous Hydrogen Peroxide in *Escherichia coli*

**DOI:** 10.3390/antiox12040786

**Published:** 2023-03-23

**Authors:** Xiangzhe Zhou, Feng Liu, Nuomin Li, Yongqian Zhang

**Affiliations:** 1 School of Life Science, Beijing Institute of Technology, Beijing 100081, China; 2 Institute of Engineering Medicine, School of Medical Technology, Beijing Institute of Technology, Beijing 100081, China

**Keywords:** dityrosine crosslinking, hydrogen peroxide, label-free proteomics

## Abstract

Excessive hydrogen peroxide causes oxidative stress in cells. The oxidation of two tyrosine residues in proteins can generate *o,o′*-dityrosine, a putative biomarker for protein oxidation, which plays critical roles in a variety of organisms. Thus far, few studies have investigated dityrosine crosslinking under endogenous or exogenous oxidative conditions at the proteome level, and its physiological function remains largely unknown. In this study, to investigate qualitative and quantitative dityrosine crosslinking, two mutant *Escherichia coli* strains and one mutant strain supplemented with H_2_O_2_ were used as models for endogenous and exogenous oxidative stress, respectively. By integrating high-resolution liquid chromatography—mass spectrometry and bioinformatic analysis, we created the largest dityrosine crosslinking dataset in *E. coli* to date, identifying 71 dityrosine crosslinks and 410 dityrosine loop links on 352 proteins. The dityrosine-linked proteins are mainly involved in taurine and hypotaurine metabolism, citrate cycle, glyoxylate, dicarboxylate metabolism, carbon metabolism, etc., suggesting that dityrosine crosslinking may play a critical role in regulating the metabolic pathways in response to oxidative stress. In conclusion, we have reported the most comprehensive dityrosine crosslinking in *E. coli* for the first time, which is of great significance in revealing its function in oxidative stress.

## 1. Introduction

Endogenous protein crosslinking is regarded as the dark matter in the proteome. Its formation affects the structure, conformation, and function of the proteins. The main endogenous protein crosslinking currently discovered in prokaryotes and eukaryotes include NOS bridge, isopeptide bond, disulfide bond, ditryptophan, formaldehyde, methylglyoxal, and dityrosine crosslinking, etc. For example, a novel protein crosslinking formed by an NOS bridge between lysine and cysteine could act as a redox switch to regulate enzyme function [1]. The presence of isopeptide bond crosslinking has been reported in 30% of spore shell proteins in *Bacillus subtilis* [2]. The oxidation of cysteine may result in a disulfide bond formation in the proteins, including crosslinked, loop linked, and complex forms. In the periplasmic fraction of *Escherichia coli*, 199 disulfide bonds were identified at the proteome level [3].

Two molecules of tyrosine residues of protein can generate stable and covalent *o,o′*-dityrosine crosslinking, a natural posttranslational modification [4], which is a putative biomarker for protein oxidation [5]. The formation of dityrosine crosslinking can be either enzyme-catalyzed [6] or non-enzyme-catalyzed [4]. Dityrosine is found in structural elements of some species [7], such as the resilin of *Schistocera gregaria* [8] and bovine skin [9]. It has been reported that dityrosine crosslinking of matrix fibronectin can resist proteolysis by multiple proteases and inhibit cellular migration [10]. Dityrosine crosslinking in amyloid plaques was increased in cerebrospinal fluid in Alzheimer’s disease (AD) patients, and it might play an important role in the pathogenesis of AD [11]. Despite tremendous achievements having been made in clarifying the dityrosine crosslinking in specific proteins, few studies have investigated dityrosine crosslinking at the proteome level under endogenous or exogenous oxidative conditions thus far.

Considering that endogenous protein crosslinking affects protein conformation and function, we hypothesized that the global analysis of dityrosine crosslinking in *E. coli* will provide insight into oxidative damage and could yield a novel concept of a dityrosine crosslinking biomarker for redox imbalance. To test this hypothesis, in this study, two mutant strains (*E. coli* MG1655/Δ*Ahp* and *E. coli* MG1655/Δ*Ahp*Δ*KatE*Δ*KatG*) preserved in our laboratory were used as endogenous oxidative stress models [12,13], and *E. coli* MG1655/Δ*Ahp*Δ*KatE*Δ*KatG* was treated with 1 mM H_2_O_2_ as an exogenous oxidative stress model. Alkyl hydroperoxide reductase (AhP), catalase G (KatG), and catalase E (KatE) are the three predominant hydrogen peroxide-degrading enzymes in *E. coli* [14]. In our previous studies, the concentrations of hydrogen peroxide in *E. coli* MG1655/Δ*Ahp* and *E. coli* MG1655/Δ*Ahp*Δ*KatE*Δ*KatG* were measured at 0.13 μM and 0.31 μM [13]. Intracellular hydrogen peroxide plays critical roles in cellular signaling and pathogen defense [15]. As a double-edged sword, excessive amounts of hydrogen peroxide have deleterious effects on cells, such as oxidative damage and redox imbalance [16]. We integrated label-free LC–MS/MS and bioinformatic tools to identify and quantify dityrosine crosslinking in endogenous and exogenous oxidative stress models. A large-scale dataset of dityrosine crosslinking in *E. coli* was created for the first time. The findings of this study suggest that dityrosine crosslinking not only could be regarded as an emerging biomarker used either alone or in a multiple biomarker panel for oxidative damage but also may play a critical role in regulating metabolic pathways in response to oxidative stress.

## 2. Materials and Methods

### 2.1. Strains and Growth Conditions

The mutant strains *E. coli* MG1655/Δ*Ahp* and *E. coli* MG1655/Δ*Ahp*Δ*KatE*Δ*KatG* are preserved by our laboratory, and the construction of the mutant strains of *E. coli* MG1655 was reported in the previous study [13]. The two mutant strains, as well as *E. coli* MG1655/Δ*Ahp*Δ*KatE*Δ*KatG* supplemented with 1 mM H_2_O_2_, were cultured in sterilized LB liquid medium at 37 °C, with shaking at 150 rpm overnight. Three strains were grown to the exponential phase (OD600 ~0.8) and then harvested. For proteomic analysis, each strain was prepared in six biological replicates.

### 2.2. Protein Extraction, Digestion, and Desalting

Every bacterial sample was centrifuged, and the pellet was collected and washed with 50 mM PBS. The bacterial pellet was resuspended in lysis buffer (8 M urea, 2 mM EDTA, 1 mM Phenylmethanesulfonyl fluoride, 50 mM NH_4_HCO_3_). Then, the cells were disrupted in an ice bath by sonication for 8 min. After that, the sample was centrifuged, and the supernatant was transferred into a new tube. The protein concentration was measured by the BCA protein method. Approximately 100 μg protein was reduced with 10 mM DTT for 30 min at 56 °C, alkylated with 50 mM iodoacetamide in the dark for 30 min, and then diluted and digested for 16 h at 37 °C by trypsin at an enzyme/protein ratio of 1:50. The residual trypsin activity was quenched by the addition of 3% formic acid (*v*/*v*). The peptides were desalted using a C_18_ solid-phase extraction (SPE) column and dried using a vacuum centrifuge [13]. The peptide concentration was determined by a BCA peptide assay.

### 2.3. LC–MS/MS Analysis

The peptide mixture was dissolved in water containing 0.1% FA and analyzed using an online U3000-nano coupled with an Orbitrap Q-Exactive HFX mass spectrometer (ThermoFisher Scientific, Waltham, MA, USA). The peptides were separated using a 15 cm homemade C_18_ reversed-phase column (100-μm inner diameter, 1.9 μm resin) and a 110 min elution gradient. Mobile phase A consisted of 0.1% FA and H_2_O, and mobile phase B consisted of 20% H_2_O and 80% ACN. A 110 min gradient (mobile phase B: 5% at 0 min, 5% at 4 min, 10% at 20 min, 22% at 64 min, 35% at 94 min, 99% at 99 min, 99% at 104 min, 5% at 105 min) was used at a flow rate of 300 nL/min. The data were acquired in a data-dependent mode. For mass spectrometry parameters, the scan range was set to 350–2000 *m*/*z*. The top 20 most intense ions in MS1 were selected for MS/MS analysis, and the dynamic exclusion time was 45 s.

### 2.4. Data Analysis

The raw mass spectrometry files from the three groups (*E. coli* MG1655/Δ*Ahp*, *E. coli* MG1655/Δ*Ahp*Δ*KatE*Δ*KatG,* and *E. coli* MG1655/Δ*Ahp*Δ*KatE*Δ*KatG* with 1 mM H_2_O_2_) were searched against the UniProt *E. coli* database (2019/10/29, taxonomy ID: 83333, 4391 sequences) concatenated with a reverse decoy database by pLink 2 for qualitative analysis of dityrosine crosslinking, respectively. The flow type was conventional crosslinking (Higher-Energy Collisional Dissociation, HCD), and the false discovery rate (FDR) was less than 0.05 at the PSM level. The alpha and beta sites of dityrosine crosslinking were both set as tyrosine (Y), and the linker mass and linker composition were −2.016 and H (−2), respectively. The following parameters were used: 20 ppm precursor tolerance, 20 ppm fragment tolerance; trypsin enzyme specificity, a maximum of three missed cleavages; fixed modification: carbamidomethyl (C), variable modification: oxidation (M).

The label-free quantification algorithm in pQuant was applied for quantification among the different groups. Then, the data were imported into Perseus software for statistical analysis. The values were log-transformed with base 2, and we filtered out the rows with missing values across the different groups. The heat map was plotted after the Z-score. After categorical annotation rows and analysis of the volcano plot (S0 = 2, FDR = 0.05), the sheets were obtained. The significantly differentially dityrosine-linked peptides were defined as *p*-value < 0.05, and log_2_(foldchange) < −1 or >1, calculated as *E. coli* MG1655/Δ*Ahp*Δ*KatE*Δ*KatG*/*E. coli* MG1655/Δ*Ahp* or *E. coli* MG1655/Δ*Ahp*Δ*KatE*Δ*KatG* with 1 mM H_2_O_2_/*E. coli* MG1655/Δ*Ahp*Δ*KatE*Δ*KatG* by six biological replicates. Volcano plots were obtained based on these thresholds.

### 2.5. Bioinformatic Analysis

For the proteins which corresponded to all the dityrosine-linked peptides identified by pLink 2 in the three groups, subcellular localization, Gene Ontology, and KEGG pathway analyses were carried out. CELLO v.2.5: subCELlular LOcalization predictor was used for subcellular localization analysis. For each protein, the most-likely-location of it was focused on and utilized for the pie diagram. DAVID Bioinformatics Resources were used for functional annotation. Gene ontology (including biological process, cellular component, and molecular function) and KEGG (Kyoto encyclopedia of gene and genomes) pathway analyses of the proteins were performed with the EASE set 0.05. Data visualization of KEGG pathways was realized by R. In addition, Gene ontology analysis of the proteins which corresponded to significantly differentially dityrosine-linked peptides in volcano plots was performed.

### 2.6. Validation of Dityrosine-Crosslinked Peptides by Mass Spectrometry

The standard peptide (LVSWYDNETGYSNK) was synthesized and purified (Sangon Biotech, Shanghai, China). Then, the 0.05 g/L standard peptide was incubated with and without 0.1 μmol/L H_2_O_2_ in a 37 °C water bath for 12 h to obtain the dityrosine-crosslinked peptides. Then, each sample was analyzed successively by LC–MS/MS with the same parameters of Section 2.3. The raw mass spectrometry file was searched using pLink 2 with the following parameters: 20 ppm precursor tolerance, 20 ppm fragment tolerance; trypsin enzyme specificity, a maximum of zero missed cleavages; fixed modification: carbamidomethyl (C), variable modification: oxidation (M). The spectra of dityrosine-crosslinked peptides were visualized by pLabel.

### 2.7. Data Availability

The spectrometry proteomics data have been deposited to the ProteomeXchange Consortium via the PRIDE [17] partner repository with the dataset identifier PXD039132.

## 3. Results

**Figure 1:**
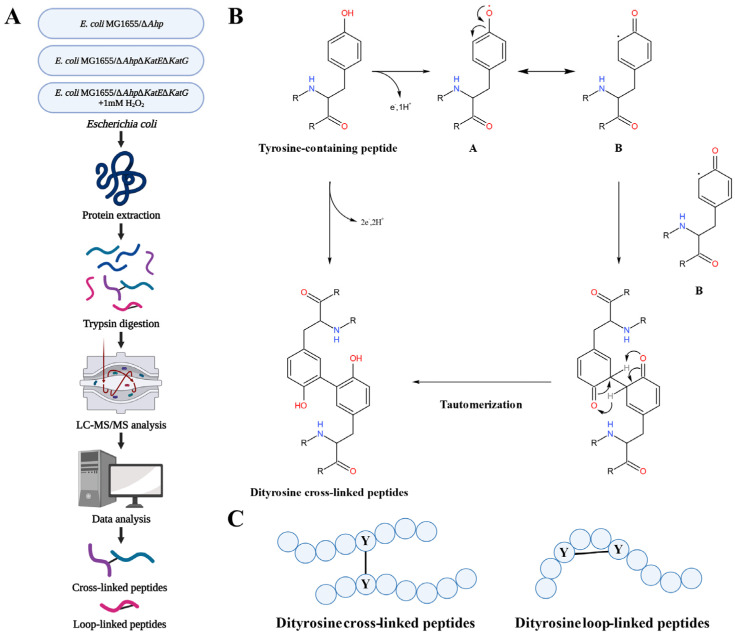
The identification, formation, and type of dityrosine crosslinking. (**A**) Experimental flowchart for the comprehensive analysis of dityrosine crosslinking in *E. coli*. The cartoon was created with BioRender.com (accessed on 6 February 2023). (**B**) The possible reaction mechanism of how dityrosine-crosslinked peptides were formed from tyrosine-containing peptides. (**C**) Schematic diagram of dityrosine-crosslinked peptides and dityrosine-loop linked peptides.

The overall experimental design is shown in Figure 1A. Two mutant strains (*E. coli* MG1655/Δ*Ahp* and *E. coli* MG1655/Δ*Ahp*Δ*KatE*Δ*KatG*) were constructed as endogenous oxidative stress models, and *E. coli* MG1655/Δ*Ahp*Δ*KatE*Δ*KatG* was treated with 1 mM H_2_O_2_ as an exogenous oxidative stress model. Two molecules of tyrosine residues of intracellular proteins can generate stable and covalent *o,o′*-dityrosine crosslinking. It should be noted that H_2_O_2_ does not react directly with tyrosine at any significant rate. It is likely that in vivo, the formation of dityrosine occurs via Fenton-like reactions. Hydroxyl radicals are a type of free radical and can be formed from hydrogen peroxide in the presence of copper or iron in vivo. The hydroxyl radicals further attack the tyrosine to generate dityrosine links within the proteins or between two different proteins in *E. coli*. The possible reaction mechanism of how dityrosine-crosslinked peptides were formed from tyrosine-containing peptides [18] is shown in Figure 1B. There were two main forms of dityrosine crosslinking in the digested peptides: dityrosine-crosslinked peptides between two different peptides and dityrosine-loop linked peptides in one linear peptide, as shown in Figure 1C.

### 3.1. Qualitative Analysis of Dityrosine Crosslinking

**Figure 2:**
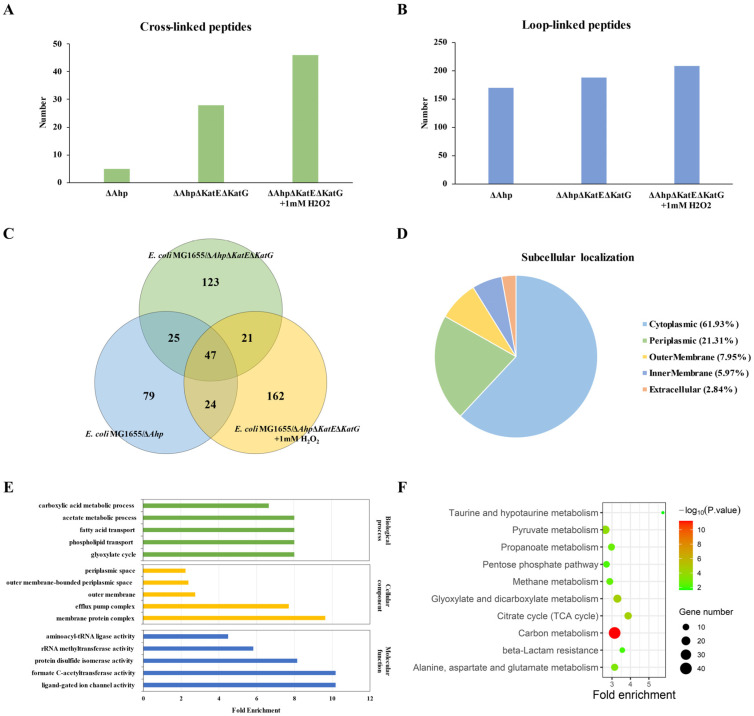
Bioinformatic analysis for qualitative data of dityrosine-linked peptides. The number of dityrosine crosslinked peptides (**A**) and -loop linked peptides (**B**) identified by pLink in the three groups (*E. coli* MG1655/Δ*Ahp*, *E. coli* MG1655/Δ*Ahp*Δ*KatE*Δ*KatG,* and *E. coli* MG1655/Δ*Ahp*Δ*KatE*Δ*KatG* supplemented with 1 mM H_2_O_2_). (**C**) Venn diagram of dityrosine-linked peptides for the three groups. (**D**) Subcellular localization of the proteins which corresponded to all the dityrosine-linked peptides identified by pLink in the three groups. (**E**) Gene ontology (TOP5 of fold enrichment for biological process, cellular component, and molecular function) analysis for all these proteins. (**F**) The TOP 10 enriched KEGG pathways for all these proteins along with *p*-value and gene number. The color represents the significance of enrichment.

The qualitative data are shown in Table S1 (*E. coli* MG1655/Δ*Ahp*), Table S2 (*E. coli* MG1655/Δ*Ahp*Δ*KatE*Δ*KatG*), and Table S3 (*E. coli* MG1655/Δ*Ahp*Δ*KatE*Δ*KatG* with 1 mM H_2_O_2_). The number of dityrosine-crosslinked peptides and -loop linked peptides identified by pLink in the three groups (*E. coli* MG1655/Δ*Ahp*, *E. coli* MG1655/Δ*Ahp*Δ*KatE*Δ*KatG,* and *E. coli* MG1655/Δ*Ahp*Δ*KatE*Δ*KatG* with 1 mM H_2_O_2_) is shown in Figure 2A,B, respectively. Each number referred to how many dityrosine-crosslinked peptides or -loop linked peptides in total, including six biological replicates for each group. As mentioned in the introduction, the concentrations of hydrogen peroxide in *E. coli* MG1655/Δ*Ahp* and *E. coli* MG1655/Δ*Ahp*Δ*KatE*Δ*KatG* were approximately 0.13 μM and 0.31 μM, respectively. It was observed that the number of ditryosine-crosslinked and -loop linked peptides elevated with increasing concentrations of intracellular hydrogen peroxide. 

A three-circle Venn diagram of dityrosine-linked peptides was used to visualize the overlap of the three groups (Figure 2C). A total of 71 dityrosine crosslinks and 410 dityrosine loop links were identified in the three groups, which might be putative biomarkers of oxidation damage and redox imbalance. The dityrosine-crosslinked and -loop linked peptides corresponded to a total of 352 proteins. Subcellular localization, gene ontology, and KEGG pathway analyses were performed based on the bioinformatic tools in Figure 2D–F. The subcellular localization included cytoplasmic (61.93%), periplasmic (21.31%), outer membrane (7.95%), inner membrane (5.97%), and extracellular (2.84%) localization. Gene ontology (TOP5 of fold enrichment for biological process, cellular component, and molecular function) analysis for all these proteins is shown in Figure 2E. The top 5 enriched biological processes were the glyoxylate cycle, phospholipid transport, fatty acid transport, acetate metabolic process, and carboxylic acid metabolic process. The top 5 enriched cellular components were the membrane protein complex, efflux pump complex, outer membrane, outer membrane-bound periplasmic space, and periplasmic space. The top 5 enriched molecular functions were ligand-gated ion channel activity, formate C-acetyltransferase activity, protein disulfide isomerase activity, rRNA methyltransferase activity, and aminoacyl-tRNA ligase activity. KEGG pathway analysis for all these proteins is shown in Figure 2F. The top 10 enriched KEGG pathways were taurine and hypotaurine metabolism; citrate cycle (TCA cycle), beta-lactam resistance, glyoxylate, and dicarboxylate metabolism; carbon metabolism; alanine, aspartate, and glutamate metabolism; propanoate metabolism; methane metabolism; pentose phosphate pathway and pyruvate metabolism.

### 3.2. Quantitative Analysis of Dityrosine Crosslinking

**Figure 3:**
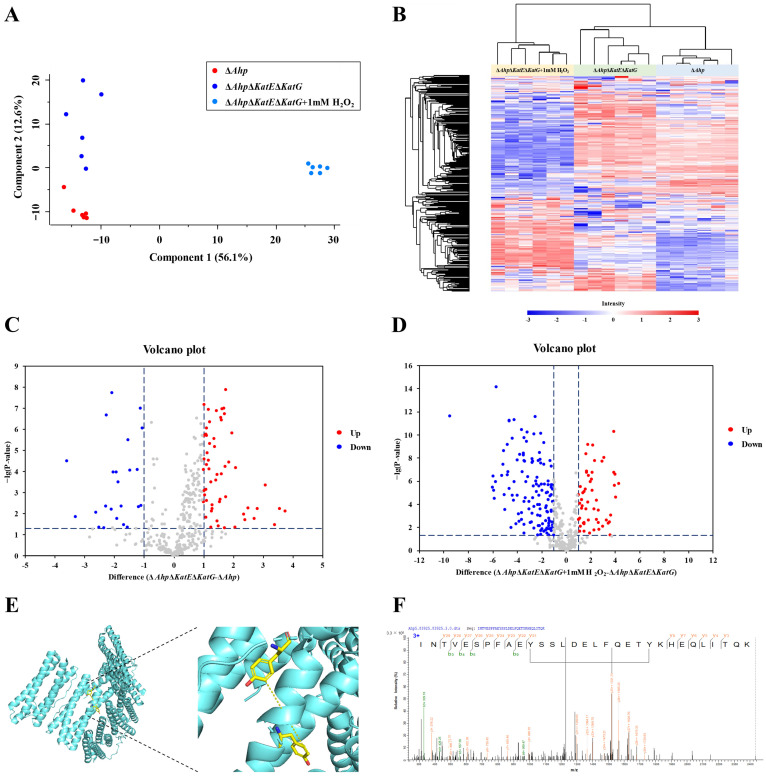
Quantification of dityrosine-linked peptides. (**A**) Principal component analysis (PCA) of the three groups. Components 1 and 2 are shown. The red dots represent *E. coli* MG1655/Δ*Ahp*, the blue dots represent *E. coli* MG1655/Δ*Ahp*Δ*KatE*Δ*KatG*, and the light blue dots represent *E. coli* MG1655/Δ*Ahp*Δ*KatE*Δ*KatG* supplemented with 1 mM H_2_O_2_. (**B**) Heatmap of the three groups, showing the differences among each group, which suggested a reliable quantification result. (**C**) Volcano plot for *E. coli* MG1655/Δ*Ahp*Δ*KatE*Δ*KatG* and *E. coli* MG1655/Δ*Ahp*. (**D**) Volcano plot for *E. coli* MG1655/Δ*Ahp*Δ*KatE*Δ*KatG* supplemented with 1 mM H_2_O_2_ and *E. coli* MG1655/Δ*Ahp*Δ*KatE*Δ*KatG*. The red dots and blue dots represent upregulated and downregulated dityrosine-linked peptides, respectively. (**E**) The location of the dityrosine-loop linked peptide is shown in yellow in the bacterial non-heme ferritin spatial structure. The 3D graph was generated from the PyMol Molecular Graphics System, and the PDB file (accession codes:1EUM) was downloaded from the RCSB website. (**F**) The MS/MS spectrum of the dityrosine-loop linked peptides in bacterial non-heme ferritin.

Label-free quantification was performed for the dityrosine crosslinking among the different groups. The quantitative data are shown in Table S4. Principal component analysis was performed to acquire a general overview of the data quality. As shown in Figure 3A, the results revealed that the dityrosine-linked peptides of each group clustered tightly and were distinct from other groups at the dityrosine-linked peptide level. This result suggested that the dityrosine-linked peptides had an outstanding performance in discriminating between the two endogenous oxidative stress models and the exogenous oxidative stress model. Moreover, the heatmap revealed that the differences in dityrosine-linked peptides among the three groups were significant (Figure 3B). A volcano plot for *E. coli* MG1655/Δ*Ahp*Δ*KatE*Δ*KatG* and *E. coli* MG1655/Δ*Ahp* is shown in Figure 3C, and a volcano plot for *E. coli* MG1655/Δ*Ahp*Δ*KatE*Δ*KatG* with 1 mM H_2_O_2_ and *E. coli* MG1655/Δ*Ahp*Δ*KatE*Δ*KatG* is shown in Figure 3D. The red dots and blue dots represent upregulated and downregulated dityrosine-linked peptides, respectively. When comparing *E. coli* MG1655/Δ*Ahp*Δ*KatE*Δ*KatG* and *E. coli* MG1655/Δ*Ahp* (Figure 3C), 23 groups of dityrosine-linked peptides were downregulated, and 58 groups of dityrosine-linked peptides were upregulated in *E. coli* MG1655/Δ*Ahp*Δ*KatE*Δ*KatG*. When comparing *E. coli* MG1655/Δ*Ahp*Δ*KatE*Δ*KatG* with 1 mM H_2_O_2_ and *E. coli* MG1655/Δ*Ahp*Δ*KatE*Δ*KatG* (Figure 3D), 130 groups of dityrosine-linked peptides were downregulated and 55 groups of dityrosine linked peptides were upregulated in *E. coli* MG1655/Δ*Ahp*Δ*KatE*Δ*KatG* with 1 mM H_2_O_2_. Among these linked peptides, one dityrosine-loop linked peptide, corresponding to bacterial non-heme ferritin, was downregulated in *E. coli* MG1655/Δ*Ahp*Δ*KatE*Δ*KatG* with 1 mM H_2_O_2_, when comparing *E. coli* MG1655/Δ*Ahp*Δ*KatE*Δ*KatG* and *E. coli* MG1655/Δ*Ahp*. The 80th and 91st tyrosine residues were loop linked and generated *o,o′*-dityrosine in bacterial non-heme ferritin, Figure 3E, and the MS/MS spectrum of the loop linked peptide is shown in Figure 3F.

**Figure 4:**
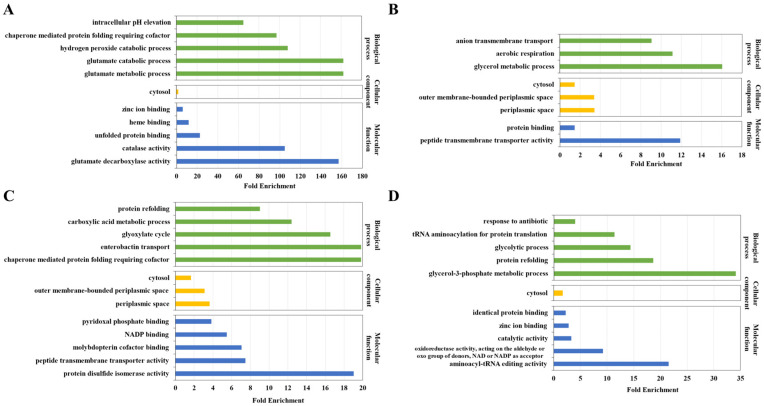
Gene ontology analysis for the proteins corresponding to the down/upregulated dityrosine-linked peptides. (**A**) Gene ontology (biological process (TOP5 of fold enrichment), cellular component, and molecular function (TOP5 of fold enrichment)) analysis for the proteins which corresponded to the dityrosine-linked peptides downregulated in *E. coli* MG1655/Δ*Ahp*Δ*KatE*Δ*KatG,* when comparing *E. coli* MG1655/Δ*Ahp*Δ*KatE*Δ*KatG* and *E. coli* MG1655/Δ*Ahp*. (**B**) Gene ontology (biological process, cellular component, and molecular function) analysis for the proteins which corresponded to the dityrosine-linked peptides upregulated in *E. coli* MG1655/Δ*Ahp*Δ*KatE*Δ*KatG,* when comparing *E. coli* MG1655/Δ*Ahp*Δ*KatE*Δ*KatG* and *E. coli* MG1655/Δ*Ahp*. (**C**) Gene ontology (biological process (TOP5 of fold enrichment), cellular component, and molecular function (TOP5 of fold enrichment)) analysis for the proteins which corresponded to the dityrosine-linked peptides downregulated in *E. coli* MG1655/Δ*Ahp*Δ*KatE*Δ*KatG* with 1 mM H_2_O_2_, when comparing *E. coli* MG1655/Δ*Ahp*Δ*KatE*Δ*KatG* with 1 mM H_2_O_2_ and *E. coli* MG1655/Δ*Ahp*Δ*KatE*Δ*KatG*. (**D**) Gene ontology (biological process (TOP5 of fold enrichment), cellular component, and molecular function (TOP5 of fold enrichment)) analysis for the proteins which corresponded to the dityrosine-linked peptides upregulated in *E. coli* MG1655/Δ*Ahp*Δ*KatE*Δ*KatG* with 1 mM H_2_O_2_, when comparing *E. coli* MG1655/Δ*Ahp*Δ*KatE*Δ*KatG* with 1 mM H_2_O_2_ and *E. coli* MG1655/Δ*Ahp*Δ*KatE*Δ*KatG*.

To obtain functional information about differentially abundant dityrosine-linked peptides, gene ontology analysis was performed on the proteins which corresponded to the upregulated and downregulated dityrosine-linked peptides. Gene ontology (biological process (TOP5 of fold enrichment), cellular component, and molecular function (TOP5 of fold enrichment)) analysis for the proteins which corresponded to the dityrosine-linked peptides downregulated in *E. coli* MG1655/Δ*Ahp*Δ*KatE*Δ*KatG,* when comparing *E. coli* MG1655/Δ*Ahp*Δ*KatE*Δ*KatG* and *E. coli* MG1655/Δ*Ahp* are shown in Figure 4A. The top 5 enriched biological processes were the glutamate metabolic process, glutamate catabolic process, hydrogen peroxide catabolic process, chaperone-mediated protein folding requiring cofactor, and intracellular pH elevation. The cellular component was cytosol. The top 5 enriched molecular functions were glutamate decarboxylase activity, catalase activity, unfolded protein binding, heme binding, and zinc ion binding. Gene ontology (biological process, cellular component, and molecular function) analysis for the proteins which corresponded to the dityrosine-linked peptides upregulated in *E. coli* MG1655/Δ*Ahp*Δ*KatE*Δ*KatG,* when comparing *E. coli* MG1655/Δ*Ahp*Δ*KatE*Δ*KatG* and *E. coli* MG1655/Δ*Ahp* are shown in Figure 4B. The biological processes were the glycerol metabolic process, aerobic respiration, and anion transmembrane transport. The cellular components were periplasmic space, outer membrane-bounded periplasmic space, and cytosol. The molecular functions were peptide transmembrane transporter activity and protein binding.

Gene ontology (biological process (TOP5 of fold enrichment), cellular component, and molecular function (TOP5 of fold enrichment)) analysis for the proteins which corresponded to the dityrosine-linked peptides downregulated in *E. coli* MG1655/Δ*Ahp*Δ*KatE*Δ*KatG* with 1 mM H_2_O_2_, when comparing *E. coli* MG1655/Δ*Ahp*Δ*KatE*Δ*KatG* with 1 mM H_2_O_2_ and *E. coli* MG1655/Δ*Ahp*Δ*KatE*Δ*KatG* are shown in Figure 4C. The top 5 enriched biological processes were chaperone-mediated protein folding requiring cofactor, enterobactin transport, glyoxylate cycle, carboxylic acid metabolic process, and protein refolding. The cellular components were periplasmic space, outer membrane-bounded periplasmic space, and cytosol. The top 5 enriched molecular functions were protein disulfide isomerase activity, peptide transmembrane transporter activity, molybdopterin cofactor binding, NADP binding, and pyridoxal phosphate binding. Gene ontology (biological process (TOP5 of fold enrichment), cellular component, and molecular function (TOP5 of fold enrichment)) analysis for the proteins which corresponded to the dityrosine-linked peptides upregulated in *E. coli* MG1655/Δ*Ahp*Δ*KatE*Δ*KatG* with 1 mM H_2_O_2_, when comparing *E. coli* MG1655/Δ*Ahp*Δ*KatE*Δ*KatG* with 1 mM H_2_O_2_ and *E. coli* MG1655/Δ*Ahp*Δ*KatE*Δ*KatG* are shown in Figure 4D. The top 5 enriched biological processes were the glycerol-3-phosphate metabolic process, protein refolding, glycolytic process, and tRNA aminoacylation for protein translation and response to antibiotics. The cellular component was cytosol. The top 5 enriched molecular functions were aminoacyl-tRNA editing activity, oxidoreductase activity, acting on the aldehyde or oxo group of donors, NAD or NADP as an acceptor, catalytic activity, zinc ion binding, and identical protein binding.

### 3.3. Validation of Dityrosine Crosslinked Peptides In Vitro

**Figure 5:**
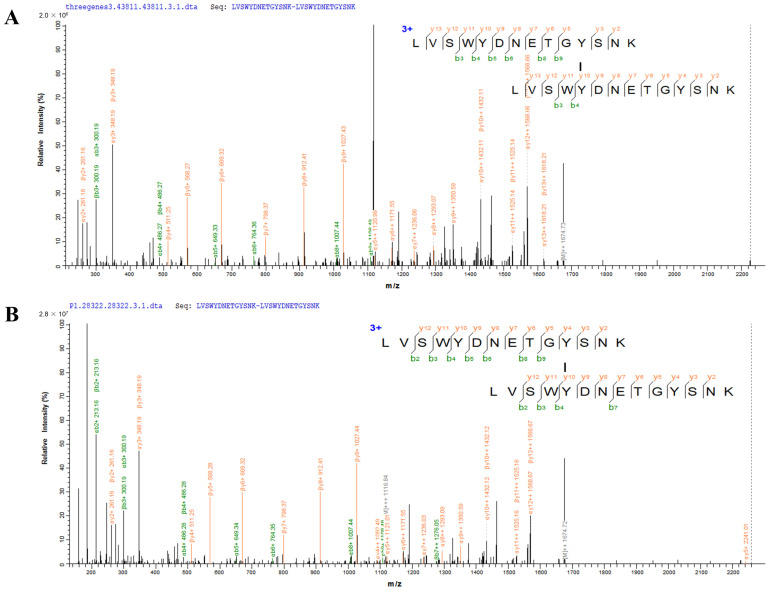
The MS/MS spectrum of dityrosine-crosslinked peptides (LVSWYDNETGYSNK-LVSWYDNETGYSNK). (**A**) The spectrum of endogenous dityrosine-crosslinked peptides (LVSWYDNETGYSNK-LVSWYDNETGYSNK) corresponding to glyceraldehyde-3-phosphate dehydrogenase A in *E. coli* MG1655/Δ*Ahp*Δ*KatE*Δ*KatG*. (**B**) The MS/MS spectrum of dityrosine-crosslinked peptides (LVSWYDNETGYSNK-LVSWYDNETGYSNK) from in vitro incubation.

To further examine the accuracy of the spectra identified by pLink, we synthesized the standard peptide (LVSWYDNETGYSNK) and obtained the dityrosine-crosslinked peptides in vitro. As shown in Figure 5A, the typical spectrum of dityrosine-crosslinked peptides corresponding to endogenous glyceraldehyde-3-phosphate dehydrogenase A in *E. coli* MG1655/Δ*Ahp*Δ*KatE*Δ*KatG* was identified by mass spectrometry. The highly reliable spectrum of the dityrosine-crosslinked peptides from in vitro incubation is shown in Figure 5B. The result was consistent with the spectral pattern obtained for the endogenous dityrosine-crosslinked peptides in Figure 5A, which suggested that the identification of the dityrosine-crosslinked peptides was correct by pLink.

## 4. Discussion

Dityrosine crosslinks are formed by two molecules of tyrosine residues of protein, which affects the structure, conformation, and function of the proteins [19]. In present study, three mutant *E. coli* strains with various H_2_O_2_ concentrations were used to investigate dityrosine crosslinking. By integrating high-resolution liquid chromatography—mass spectrometry, and bioinformatic analysis, we identified 71 dityrosine crosslinks and 410 dityrosine loop links on 352 proteins in *E. coli*. 

A number of assays have been developed to measure dityrosine crosslinks. For example, the presence of dityrosine was observed by fluorescence microscope in *Candida albicans* cell walls [20]. Dityrosine was also quantified by UV absorbance and fluorescence measurement in the plaques of Alzheimer’s patients [21]. Moreover, rabbit polyclonal and mouse monoclonal antibodies have been developed to detect the dityrosine level in lipofuscin granules in aged human brains [22] and in atherosclerotic lesions in mice [23], respectively. Despite advances in the identification of dityrosine crosslinks, the large-scale and precise identification of dityrosine sites on proteins remains to be addressed. The rapid development of mass spectrometry technologies and approaches enabled the identification of dityrosine crosslinks at the proteome level. Therefore, the label-free endogenous crosslinking omics strategy was performed to identify and quantify dityrosine crosslinks in *E. coli*.

In this study, two mutant *Escherichia coli* strains and one mutant strain supplemented with H_2_O_2_ were used as models for endogenous and exogenous oxidative stress, respectively. The results of this study demonstrated that dityrosine crosslinking was found in several outer membrane proteins, such as TolC and OmpA. We also observed that the dityrosine-crosslinked peptide (478th–485th) in chaperone GroEL was downregulated in *E. coli* MG1655/Δ*Ahp*Δ*KatE*Δ*KatG* compared with *E. coli* MG1655/Δ*Ahp*, which may affect the binding ability of its ATP binding site [24]. The dityrosine-crosslinked peptide (80th–91st) in bacterial non-heme ferritin was also downregulated, which may affect the binding of Fe^2+^ to this protein [25]. As mentioned before, the crosslinking formation could affect the protein conformation and function. A previous study demonstrated that tyrosine crosslinking and oxidation could result in structural and functional changes in RNAse A [26]. It was also worth noting that dityrosine levels are associated with aging, neurodegenerative diseases [27], acute inflammation [28], and atherosclerosis [29,30]. The dityrosine level was elevated in the urine of both elderly rats and patients [31,32]. Dityrosine was expected to evaluate plasma redox status in patients with hyperlipidemia [33]. All these findings uncovered that dityrosine crosslinking might be a promising biomarker for diseases associated with oxidative stress. 

Although 71 dityrosine crosslinks and 410 dityrosine loop links on 352 proteins were identified in this study, illuminating the dityrosine crosslinking and corresponding function still face great challenges. One possible problem is that the abundance of native crosslinking in the cell is extremely low (Figure S1), which is difficult to detect by mass spectrometry. Therefore, the development of enrichment, fractionation, and data acquisition assays for crosslinking omics in wet laboratories is urgent. Furthermore, qualitative and quantitative analysis and machine-learning-based bioinformatic software can be considered a subsequent priority in dry laboratories, which would help identify more protein crosslinking and quantify more accurately.

## 5. Conclusions

The number and function of endogenous dityrosine crosslinking has been underestimated in *Escherichia coli* thus far. In this study, to investigate qualitative and quantitative dityrosine crosslinking, two mutant strains were utilized as endogenous oxidative stress models, and one mutant strain supplemented with 1 mM H_2_O_2_ as an exogenous oxidative stress model. We integrated label-free LC–MS/MS and bioinformatic analysis to create the largest dityrosine crosslinking dataset in *E. coli* to date, identifying 71 dityrosine crosslinks and 410 dityrosine loop links on 352 proteins. Quantitative analysis of dityrosine crosslinking indicated that two endogenous oxidative stress models and the exogenous oxidative stress model could be discriminated at the dityrosine-linked peptide level. The findings of this study demonstrate that dityrosine crosslinking not only may play a critical role in regulating metabolic pathways in response to oxidative stress but also provide new insights into oxidative damage. It also yields emerging dityrosine-crosslinked biomarkers for redox imbalance. Taken together, we report the most comprehensive dityrosine crosslinking dataset in *E. coli* for the first time, which is of great significance in revealing its function in oxidative stress.

## Data Availability

The spectrometry proteomics data have been deposited to the ProteomeXchange Consortium via the PRIDE partner repository with the dataset identifier PXD039132.

## Supplementary Materials

The following supporting information can be downloaded at: https://www.mdpi.com/article/10.3390/antiox12040786/s1, Figure S1: A scatter diagram for comparison of the intensity of peptides and dityrosine-linked peptides. The blue dots represent the intensity of peptides, and the red dots represent the intensity of dityrosine-linked peptides. Table S1: The list of dityrosine-crosslinked peptides and -loop linked peptides identified by pLink in *E. coli* MG1655/ΔAhp group. Table S2: The list of dityrosine-crosslinked peptides and -loop linked peptides identified by pLink in *E. coli* MG1655/ΔAhpΔKatEΔKatG group. Table S3: The list of dityrosine-crosslinked peptides and -loop linked peptides identified by pLink in *E. coli* MG1655/ΔAhpΔKatEΔKatG with 1 mM H_2_O_2_ group. Table S4: The quantitative results of dityrosine-crosslinked peptides and -loop linked peptides in three groups.
