# Supplementary figures and images for: Large-Scale Qualitative and Quantitative Assessment of Dityrosine Crosslinking Omics in Response to Endogenous and Exogenous Hydrogen Peroxide in Escherichia coli
Source: Antioxidants (Basel). 2023 Mar 23;12(4):786. doi: 10.3390/antiox12040786 (PMC10135038; doi:10.3390/antiox12040786)

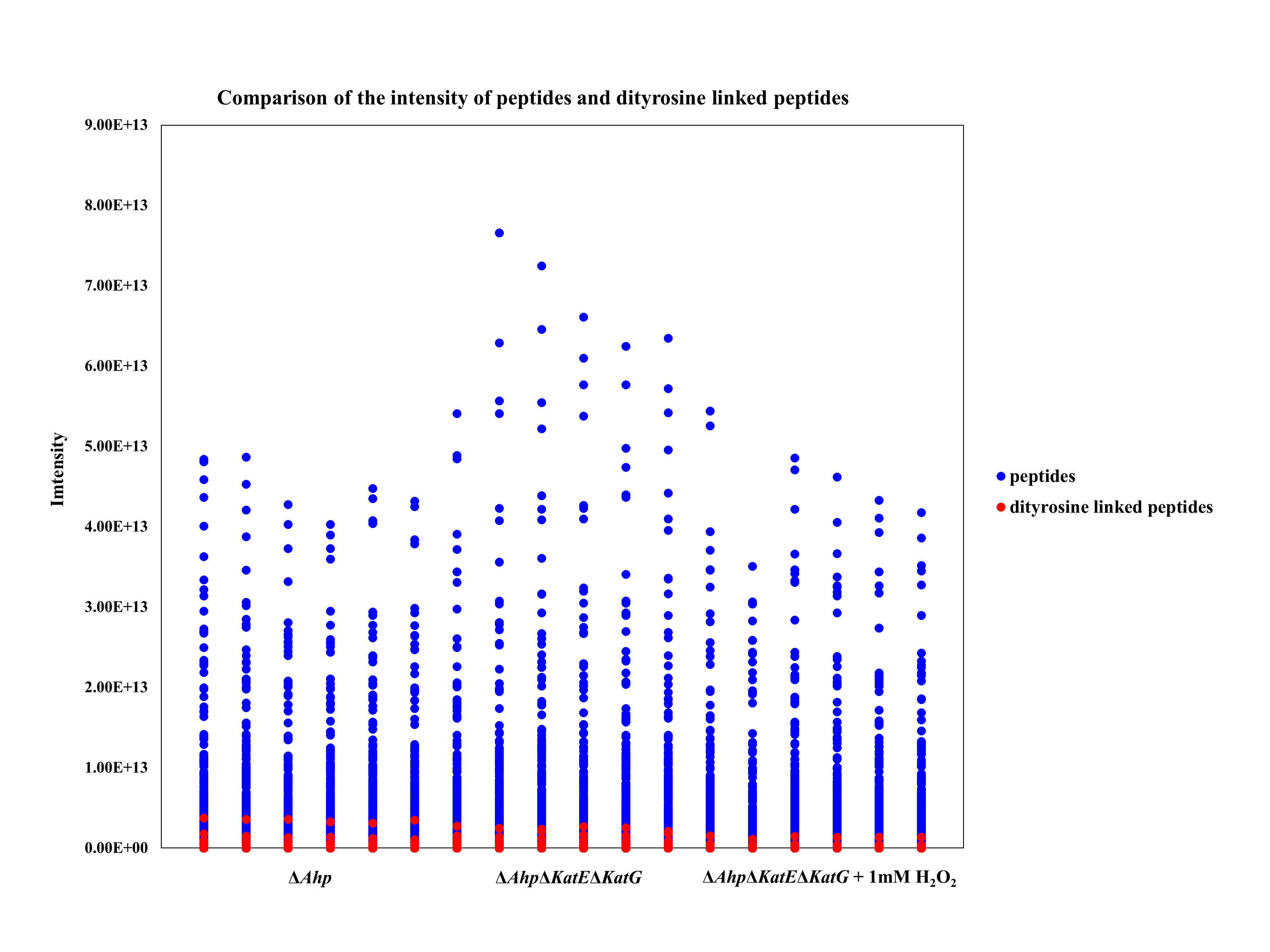

Supplement: Supplementary file 1 [file antioxidants-12-00786-s001.zip › Figure S1.tif]
